# Association of systolic blood pressure variability with cognitive decline in type 2 diabetes: A post hoc analysis of a randomized clinical trial

**DOI:** 10.1111/1753-0407.70020

**Published:** 2024-10-29

**Authors:** Junmin Chen, Xuan Zhao, Huidan Liu, Kan Wang, Xiaoli Xu, Siyu Wang, Mian Li, Ruizhi Zheng, Libin Zhou, Yufang Bi, Yu Xu

**Affiliations:** ^1^ School of Integrative Medicine Shanghai University of Traditional Chinese Medicine Shanghai China; ^2^ Department of Endocrine and Metabolic Diseases, Shanghai Institute of Endocrine and Metabolic Diseases, Ruijin Hospital Shanghai Jiaotong University School of Medicine Shanghai China; ^3^ National Clinical Research Center for Metabolic Diseases (Shanghai), Key Laboratory for Endocrine and Metabolic Diseases of the National Health Commission, National Research Center for Translational Medicine, State Key Laboratory of Medical Genomics, Ruijin Hospital Shanghai Jiaotong University School of Medicine Shanghai China

**Keywords:** blood pressure variability, cognitive function, type 2 diabetes

## Abstract

**Background:**

We aimed to explore the association between visit‐to‐visit systolic blood pressure variability (BPV) and cognitive function in individuals with type 2 diabetes.

**Methods:**

We performed a post hoc analysis of the Action to Control Cardiovascular Risk in Diabetes Memory in Diabetes (ACCORD‐MIND) substudy. A total of 2867 diabetes patients with ≥3 BP measurements between the 4‐ and 20‐month visits were included. Visit‐to‐visit systolic BPV was calculated. Cognitive decline was defined as a Mini‐Mental State Exam (MMSE), Digit Symbol Substitution Test (DSST), or Rey Auditory Verbal Learning Test (RAVLT) score greater than 1 standard deviation (SD) below the baseline mean, or a Stroop test score more than 1 SD above the baseline mean. The associations of systolic BPV with risks of cognitive decline were examined using Cox proportional hazards models, and with changes in brain magnetic resonance imaging parameters were evaluated using mixed models.

**Results:**

The risk of cognitive decline defined by the DSST score (but not by other scores) increased significantly with systolic BPV quartiles (*p* for trend = 0.008), and there was a 55% increased risk for BPV quartile 4 versus quartile 1 (hazard ratio = 1.55, 95% confidence interval 1.10–2.19). Furthermore, a positive correlation was observed between systolic BPV and change in white matter lesion volume (*β* = 0.07, 95% CI 0.01–0.13).

**Conclusions:**

A greater visit‐to‐visit systolic BPV was significantly associated with an increased risk of cognitive decline measured by DSST and an increase in white matter lesion volume in patients with type 2 diabetes.

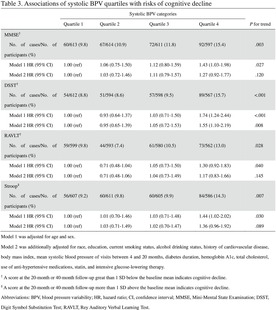

## Graphical abstract

- This post hoc analysis of the Action to Control Cardiovascular Risk in Diabetes Memory in Diabetes (ACCORD‐MIND) substudy showed that high visit‐to‐visit systolic blood pressure variability (BPV) was associated with an increased risk of cognitive decline defined by the Digit Symbol Substitution Test (DSST) score.
- Further, visit‐to‐visit systolic BPV was positively associated with change in white matter lesion volume evaluated by brain magnetic resonance imaging.
- Visit‐to‐visit systolic BPV might play a role in the decline of cognitive function in individuals with type 2 diabetes.

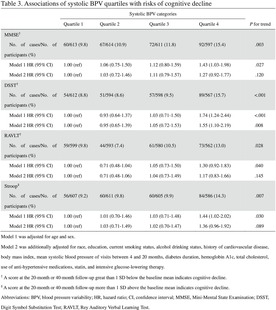

## 1 INTRODUCTION

Approximately 35 million people around the world are living with dementia, and the number is expected to be doubled by 2030 and tripled by 2050, posing a significant concern on public health.^1^, ^2^ There are currently no effective treatments available to reverse cognitive decline and dementia. Therefore, measures to prevent or slow down its progression are urgently needed.^3^

During the last 20 years, there has been an increasing body of evidence connecting elevated blood pressure (BP) and cognitive decline.^1^, ^3^, ^4^, ^5^ In addition to traditional risk factors such as hypertension, diabetes, dyslipidemia, and arterial fibrillation, variabilities in BP from visit to visit are also related to cerebrovascular damage, independent of mean BP levels.^5^ Cerebrovascular disease is an important cause of cognitive decline.^2^ Cognitive impairment associated with cerebrovascular disease may range from “focal” deficits, such as aphasia, visual agnosia, or neglect, to more general impairments including mild impairment to severe dementia. Therefore, BP variability (BPV) may also affect cognitive function. A prospective cohort study found that higher visit‐to‐visit BPV, independent of mean BP, was associated with structural brain damage and impaired cognitive function among elderly adults.^6^

The rate of whole brain atrophy as an indicator of cognitive decline progression has been demonstrated,^7^ with notable distinctions observed between individuals with and without diabetes.^8^, ^9^ Smaller total brain volumes (TBV) predicted future cognitive disorders.^10^ The magnetic resonance imaging (MRI) results indicated an abnormal volume of white matter tissue, which signifies the presence of diffuse and focal ischemic, demyelinating, and inflammatory processes that contribute to small vessel disease. This condition is commonly associated with diabetes and cognitive impairment.^9^, ^11^ Furthermore, brain microvascular disease may also result in gray matter atrophy.^12^

It is known that diabetes may increase BPV through microvascular dysfunction,^13^ which is independently associated with cognitive decline and dementia.^14^ The associations between BPV and cognitive decline in patients with diabetes remain unknown.

## 2 METHODS

### 2.1 Study design and participants

This is a post hoc analysis of the Action to Control Cardiovascular Risk in Diabetes (ACCORD) trial. There have been previously published reports on the rationale and design of the trial.^15^ It was a multicenter clinical trial that enrolled individuals who had type 2 diabetes with a glycated hemoglobin level of 7.5% or higher and who also exhibited evidence of cardiovascular disease (CVD) or significant atherosclerosis, albuminuria, left ventricular hypertrophy, or at least two additional risk factors for CVD (dyslipidemia, hypertension, smoking, or obesity). A total of 10 251 patients were randomly assigned to intensive glucose‐lowering therapy (targeting a glycated hemoglobin level <6.0%) or standard glucose‐lowering therapy (targeting a glycated hemoglobin level of 7.0% to 7.9%). Among them, 4733 patients were also randomly assigned to intensive BP‐lowering treatment to reduce systolic BP to <120 mmHg or standard BP‐lowering treatment to reduce systolic BP to <140 mmHg. BP levels were measured at baseline and every 4 months thereafter in the standard treatment group, and every 2 months in the intensive treatment group. The study protocol received approval from the institutional review boards of all participating sites, in addition to a review panel at the National Heart, Lung, and Blood Institute. Written informed consent was obtained from all patients. The current study adhered to the Strengthening the Reporting of Observational Studies in Epidemiology (STROBE) reporting guideline specifically designed for cohort studies.

**FIGURE 1:**
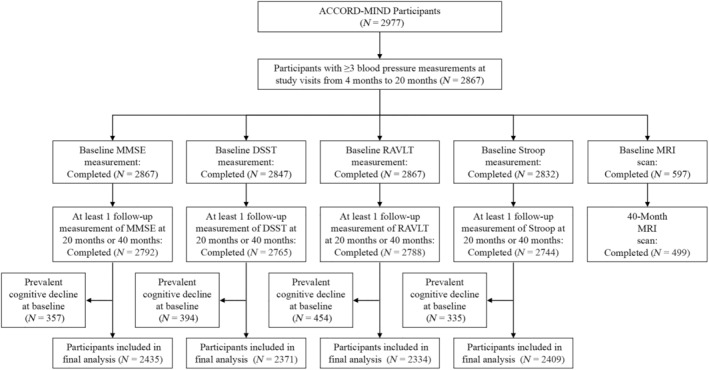
Flowchart of study participants. Prevalent cognitive decline at baseline was defined as an individual baseline MMSE, DSST, or RAVLT score greater than 1 SD below the baseline mean, or an individual baseline Stroop score more than 1 SD above the baseline mean. ACCORD‐MIND, Action to Control Cardiovascular Risk in Diabetes Memory in Diabetes; DSST, Digit Symbol Substitution Test; MMSE, Mini‐Mental State Examination; MRI, magnetic resonance imaging; RAVLT, Rey Auditory Verbal Learning Test; SD, standard deviation.

The current post hoc analysis was conducted using data from the Memory in Diabetes (MIND) substudy of the ACCORD trial,^16^ which examined the effect of intensive glucose‐lowering therapy on cognitive decline compared with standard glucose‐lowering therapy. A total of 2977 ACCORD participants participated in the ACCORD‐MIND substudy, of which 614 underwent MRI of the brain that provided useful scans.^17^ Cognitive function was measured at baseline, after 20 months, and 40 months. MRI was completed at baseline and after 40 months. We included 2867 participants who had undergone at least three BP measurements at study visits from 4 to 20 months, among whom 2792 participants completed both baseline and at least 1 follow‐up measurement of the Mini‐Mental State Exam (MMSE); 2765 participants completed both baseline and at least 1 follow‐up measurement of the Digit Symbol Substitution Test (DSST); 2788 participants completed both baseline and at least 1 follow‐up measurement of the Rey Auditory Verbal Learning Test (RAVLT); and 2744 participants completed both baseline and at least 1 follow‐up measurement of the Stroop test. Additionally, 499 participants completed both baseline and 40‐month MRI scans (Figure 1).

### 2.2 Assessments of BPV and cognitive function

The visit‐to‐visit systolic BPV was defined as the coefficient of variance in systolic BP levels at each study visit between 4 and 20 months. It was calculated as (intra‐individual standard deviation [SD] of systolic BP divided by the mean systolic BP) × 100. Systolic BP levels at the baseline visit and visits of the first 3 months were not included to exclude the impact of a rapid BP reduction during BP level titration.

The ACCORD‐MIND substudy evaluated participants' cognitive function using four scales including the MMSE, the DSST, the RAVLT, and a modified Stroop color and word test. The MMSE was used to assess an individual's global cognitive function.^18^ The DSST was used to examine psychomotor function and working memory speed.^19^ The RAVLT was used to evaluate verbal memory^20^ and the Stroop test was used to measure executive functioning.^21^ A higher score indicates better cognitive performance in the MMSE, DSST, and RAVLT tests while a higher score indicates poorer cognitive performance in the Stroop test.

In addition, MRI outcomes, including TBV, gray matter volume, white matter volume, and white matter lesion volume (reflecting both diffuse small vessel disease and the hyperintensities that surround focal lesions), were used to evaluate participants' brain structure characteristics. MRI scan protocol, image analysis, and quality control (QC) procedures have been described in detail previously.^16^, ^17^, ^22^, ^23^ Briefly, the standardized MRI scan protocol was run on 1.5‐T scanners, and an automated multi‐spectral computer program was performed to classify the brain into cerebrospinal fluid, gray matter, and white matter. White matter lesions were identified using a previously validated algorithm called white matter lesion segmentation, which is a support vector machine classifier trained on expert‐defined white matter lesions. Monthly MRI QC was managed by the MRI Reading Center following the American College of Radiology's (ACR) MRI QC Program.^24^

### 2.3 Statistical analysis

General characteristics, cognitive test scores, and brain MRI imaging parameters were presented by systolic BPV quartiles. Continuous variables were presented as means ± SDs, and categorical variables were presented as numbers and percentages. The percentage of participants with missing covariates was only 1.8%; therefore, we imputed missing covariates using the mean or median. We calculated the mean and SD of baseline scores for the MMSE, the DSST, the RAVLT, and the Stroop test among 2977 participants in the ACCORD‐MIND substudy. Cognitive decline in each cognitive domain was defined as a MMSE, DSST, or RAVLT score greater than 1 SD below the baseline mean, or a Stroop score more than 1 SD above the baseline mean.^25^

We excluded participants with baseline cognitive decline for the examination of associations between systolic BPV and subsequent cognitive decline at the 20‐ or 40‐month follow‐up visits. After excluding individuals with cognitive decline at baseline, the survival analyses included 2435, 2371, 2334, and 2409 participants for the MMSE, the DSST, the RAVLT, and the Stroop test, respectively (Figure 1). Time to first event survival curves were plotted with the Kaplan–Meier method and compared between systolic BPV quartile groups with the log‐rank test. The association between systolic BPV and the occurrence of cognitive decline was analyzed by the Cox proportional hazards model with adjustment for age, sex, race, education, current smoking status, alcohol drinking status, history of CVD, body mass index (BMI), mean systolic blood pressure (SBP) of visits between 4 and 20 months, diabetes duration, hemoglobin A1c (HbA1c), total cholesterol, use of anti‐hypertensive medications, statin, and intensive glucose‐lowering therapy. The lowest systolic BPV quartile was used as the reference group. Results were presented as hazard ratios (HRs) and 95% confidence intervals (95% CIs). We also evaluated the associations of systolic BPV with changes in four MRI measures (TBV, gray matter volume, white matter volume, and white matter lesion volume) between baseline and the 40‐month follow‐up using mixed effects models. Model 1 was adjusted for age, sex, and days since randomization, with random effects for participant. Model 2 was additionally adjusted for race, education, current smoking status, alcohol drinking status, history of CVD, BMI, mean SBP of visits between 4 and 20 months, diabetes duration, HbA1c, total cholesterol, use of anti‐hypertensive medications, statin, and intensive glucose‐lowering therapy. Results were presented as *β* (95% CI).

Statistical analysis was conducted using R software version 4.3.1. A two‐sided *p* value <0.05 was considered statistically significant.

## 3 RESULTS

### 3.1 Baseline characteristics of study participants

Characteristics of participants by quartiles of systolic BPV are described in Table 1. Participants in higher quartiles of systolic BPV were older, more likely to be male, and had higher levels of mean systolic BP and HbA1c, longer durations of diabetes, and a higher percentage of CVD history (*p* values for trend < 0.01). Baseline MMSE, DSST, and RAVLT scores decreased and Stroop test score increased significantly with increasing systolic BPV quartiles (*p* values for trend < 0.05). In addition, participants in higher quartiles of systolic BPV had lower baseline TBV, gray matter volume, and white matter volume but potentially higher white matter lesion volume than lower quartiles of systolic BPV (Table 2).

**TABLE 1:** Baseline characteristics of study participants.

Characteristics	Systolic BPV categories (*n* = 2867)				*p* for trend
	Quartile 1 (*n* = 717)	Quartile 2 (*n* = 716)	Quartile 3 (*n* = 717)	Quartile 4 (*n* = 717)	
Age (years)	62.4 ± 5.6	62.7 ± 5.8	63.2 ± 5.9	63.4 ± 5.8	<0.001
Female, *n* (%)	417 (58.2)	417 (58.2)	368 (51.3)	334 (46.6)	<0.001
Race, *n* (%)					0.009
White	517 (72.1)	506 (70.7)	514 (71.7)	467 (65.1)	
Non‐White	200 (27.9)	210 (29.3)	203 (28.3)	250 (34.9)	
Education, *n* (%)					0.005
Less than college	261 (36.4)	275 (38.4)	276 (38.5)	300 (41.8)	
Some college/technical school	255 (35.6)	227 (31.7)	257 (35.8)	257 (35.8)	
College graduate or more	201 (28.0)	214 (29.9)	184 (25.7)	160 (22.3)	
Current smoking, *n* (%)	62 (8.6)	79 (11.0)	67 (9.3)	78 (10.9)	0.317
Alcohol consumption, *n* (%)	169 (23.6)	176 (24.6)	155 (21.6)	148 (20.6)	0.093
History of CVD, *n* (%)	182 (25.4)	184 (25.7)	215 (30.0)	247 (34.4)	<0.001
BMI (kg/m^2^)	33.0 ± 5.2	32.7 ± 5.1	33.0 ± 5.3	33.2 ± 5.7	0.284
Mean SBP^a^ (mmHg)	125.6 ± 11.4	126.5 ± 12.5	127.8 ± 12.8	129.4 ± 14.5	<0.001
Mean DBP^a^ (mmHg)	70.0 ± 7.9	70.2 ± 8.4	70.1 ± 8.9	69.8 ± 8.9	0.630
Duration of diabetes (years)	9.7 ± 7.1	10.2 ± 6.8	10.4 ± 7.1	11.0 ± 7.7	<0.001
HbA1c (%)	8.2 ± 1.0	8.3 ± 1.0	8.2 ± 1.0	8.4 ± 1.1	0.006
Total cholesterol (mmol/L)	4.7 ± 1.1	4.7 ± 1.1	4.8 ± 1.1	4.7 ± 1.1	0.302
Anti‐hypertensive medications, *n* (%)	565 (78.8)	575 (80.3)	594 (82.8)	630 (87.9)	<0.001
Statin, *n* (%)	480 (66.9)	469 (65.5)	456 (63.6)	483 (67.4)	0.935
Intensive glucose‐lowering therapy, *n* (%)	354 (49.4)	338 (47.2)	352 (49.1)	362 (50.5)	0.531
MMSE	27.5 ± 2.4	27.5 ± 2.5	27.5 ± 2.5	27.2 ± 2.6	0.049
DSST	54.1 ± 15.1	53.1 ± 15.8	53.1 ± 15.8	50.5 ± 15.8	<0.001
RAVLT	7.6 ± 2.5	7.6 ± 2.5	7.5 ± 2.6	7.4 ± 2.6	0.029
Stroop	30.8 ± 14.9	31.4 ± 15.7	32.1 ± 16.1	32.7 ± 15.3	0.014

*Note*: Data are means ± standard deviations or numbers (percentages).

Abbreviations: BMI, body mass index; BPV, blood pressure variability; CVD, cardiovascular disease; DBP, diastolic blood pressure; DSST, Digit Symbol Substitution Test; HbA1c, glycosylated hemoglobin A1c; MMSE, Mini‐Mental State Examination; RAVLT, Rey Auditory Verbal Learning Test; SBP, systolic blood pressure.

^a^ Mean blood pressure of visits between 4 and 20 months.

**TABLE 2:** Brain magnetic resonance imaging parameters at baseline and 40 months.

	Total (*n* = 499)	Systolic BPV categories (*n* = 499)				*p* for trend
		Quartile 1 (*n* = 104)	Quartile 2 (*n* = 135)	Quartile 3 (*n* = 137)	Quartile 4 (*n* = 123)	
Total brain volume, cm^3^						
Baseline	925.0 ± 88.8	943.5 ± 92.4	925.2 ± 84.3	926.1 ± 88.4	908.0 ± 88.7	0.004
40 months	911.6 ± 87.8	928.4 ± 90.6	911.6 ± 83.9	912.2 ± 88.2	896.8 ± 87.5	0.010
40‐month change	−13.4 ± 16.0	−15.0 ± 16.0	−13.6 ± 16.3	−13.9 ± 15.8	−11.1 ± 15.8	0.091
*p* for 40‐month change	0.017	0.238	0.003	0.569	0.027	
Gray matter volume, cm^3^						
Baseline	462.9 ± 43.9	472.3 ± 45.4	463.5 ± 43.4	463.4 ± 43.8	454.0 ± 42.0	0.003
40 months	445.8 ± 44.1	453.4 ± 45.9	446.9 ± 43.8	445.1 ± 45.1	439.1 ± 41.2	0.015
40‐month change	−17.1 ± 17.4	−18.9 ± 15.9	−16.6 ± 17.0	−18.4 ± 18.7	−14.9 ± 17.4	0.166
*p* for 40‐month change	<0.001	0.187	0.002	0.630	0.005	
White matter volume, cm^3^						
Baseline	462.1 ± 53.4	470.5 ± 55.8	461.5 ± 50.4	462.6 ± 54.0	454.9 ± 53.4	0.041
40 months	465.9 ± 54.0	474.9 ± 56.0	464.4 ± 49.4	467.2 ± 55.4	458.7 ± 54.8	0.042
40‐month change	3.9 ± 18.2	4.4 ± 17.0	2.9 ± 18.3	4.5 ± 18.0	3.8 ± 19.6	0.959
*p* for 40‐month change	0.253	0.193	<0.001	0.493	0.010	
White matter lesion volume, cm^3^						
Baseline	1.8 ± 2.5	1.6 ± 2.8	1.5 ± 2.2	1.9 ± 2.4	2.0 ± 2.6	0.111
40 months	2.7 ± 3.0	2.5 ± 3.3	2.3 ± 2.5	2.7 ± 2.9	3.1 ± 3.2	0.071
40‐month change	0.9 ± 1.2	0.9 ± 1.3	0.8 ± 0.9	0.8 ± 1.1	1.1 ± 1.5	0.264
*p* for 40‐month change	<0.001	0.322	0.005	0.587	0.002	

*Note*: Data are means ± standard deviations.

Abbreviation: BPV, blood pressure variability.

### 3.2 Association of systolic BPV with cognitive decline

**FIGURE 2:**
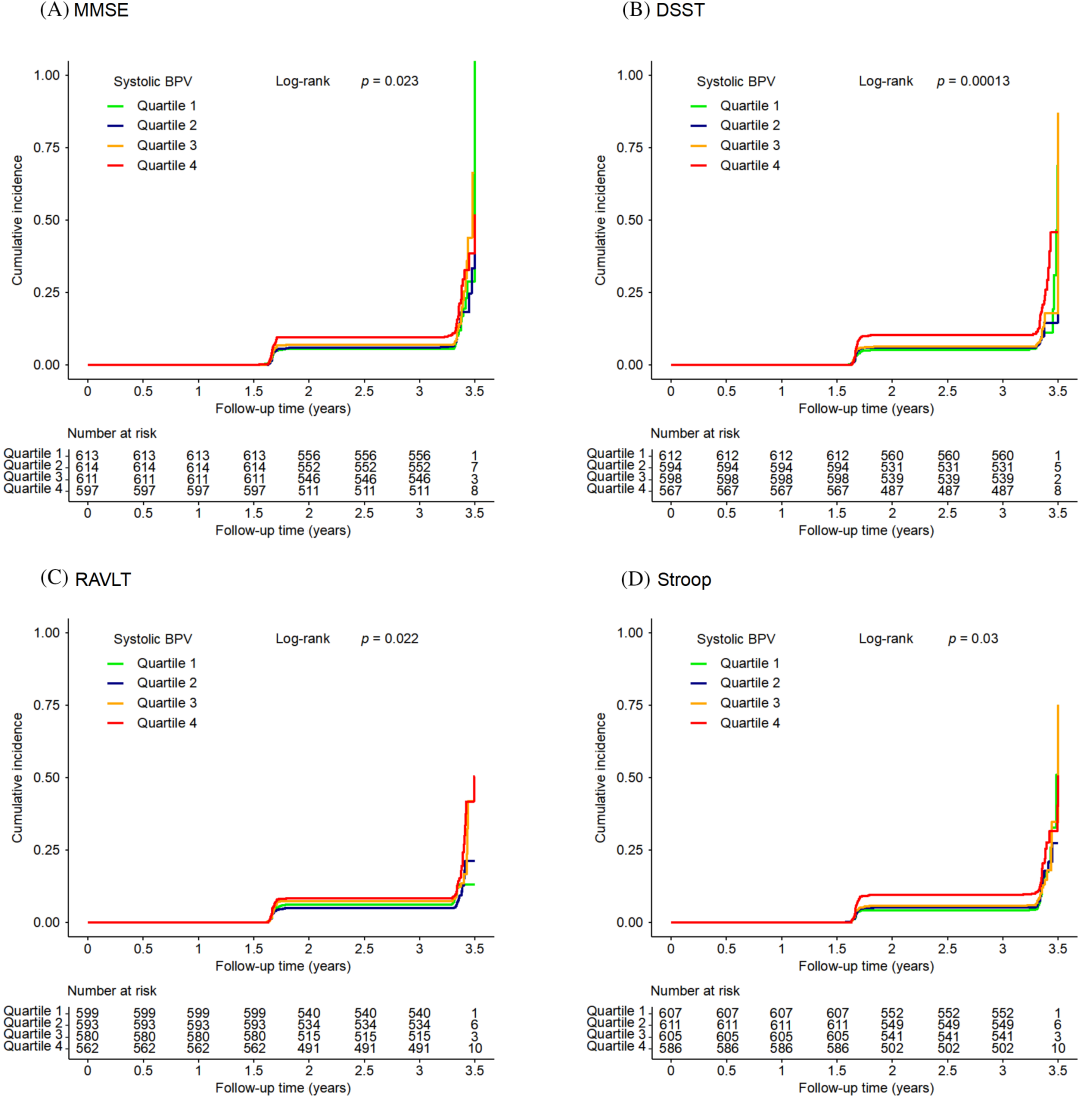
Kaplan–Meier curves for cumulative incidence of cognitive decline according to systolic BPV quartiles. BPV, blood pressure variability; DSST, Digit Symbol Substitution Test; MMSE, Mini‐Mental State Examination; RAVLT, Rey Auditory Verbal Learning Test.

Kaplan–Meier curves of the cumulative incidence of cognitive decline were plotted by quartiles of systolic BPV. There were significant differences among systolic BPV groups in cognitive decline defined by any of the four cognitive test scores (all log‐rank *p* values <0.05; Figure 2). The associations between systolic BPV and cognitive decline are shown in Table 3. The occurrence of cognitive decline defined by the DSST score increased with systolic BPV quartiles (*p* for trend = 0.008), and quartile 4 of the systolic BPV was significantly associated with an increased risk of cognitive decline defined by the DSST score (HR = 1.55, 95% CI 1.10–2.19) compared with quartile 1 of the systolic BPV after a full adjustment. No significant associations were found for systolic BPV quartiles and cognitive decline defined by the other cognitive scores after a full adjustment.

**TABLE 3:** Associations of systolic blood pressure variability (BPV) quartiles with risks of cognitive decline.

	Systolic BPV categories				*p* for trend
	Quartile 1	Quartile 2	Quartile 3	Quartile 4	
MMSE^a^					
No. of cases/no. of participants (%)	60/613 (9.8)	67/614 (10.9)	72/611 (11.8)	92/597 (15.4)	0.003
Model 1 HR (95% CI)	1.00 (ref)	1.06 (0.75–1.50)	1.12 (0.80–1.59)	1.43 (1.03–1.98)	0.027
Model 2 HR (95% CI)	1.00 (ref)	1.03 (0.72–1.46)	1.11 (0.79–1.57)	1.27 (0.92–1.77)	0.120
DSST^a^					
No. of cases/no. of participants (%)	54/612 (8.8)	51/594 (8.6)	57/598 (9.5)	89/567 (15.7)	<0.001
Model 1 HR (95% CI)	1.00 (ref)	0.93 (0.64–1.37)	1.03 (0.71–1.50)	1.74 (1.24–2.44)	<0.001
Model 2 HR (95% CI)	1.00 (ref)	0.95 (0.65–1.39)	1.05 (0.72–1.53)	1.55 (1.10–2.19)	0.008
RAVLT^a^					
No. of cases/no. of participants (%)	59/599 (9.8)	44/593 (7.4)	61/580 (10.5)	73/562 (13.0)	0.028
Model 1 HR (95% CI)	1.00 (ref)	0.71 (0.48–1.04)	1.05 (0.73–1.50)	1.30 (0.92–1.83)	0.040
Model 2 HR (95% CI)	1.00 (ref)	0.71 (0.48–1.06)	1.04 (0.73–1.49)	1.17 (0.83–1.66)	0.145
Stroop^b^					
No. of cases/no. of participants (%)	56/607 (9.2)	60/611 (9.8)	60/605 (9.9)	84/586 (14.3)	0.007
Model 1 HR (95% CI)	1.00 (ref)	1.01 (0.70–1.46)	1.03 (0.71–1.48)	1.44 (1.02–2.02)	0.030
Model 2 HR (95% CI)	1.00 (ref)	1.03 (0.71–1.49)	1.02 (0.70–1.47)	1.36 (0.96–1.92)	0.089

*Note*: Model 1 was adjusted for age and sex. Model 2 was additionally adjusted for race, education, current smoking status, alcohol drinking status, history of cardiovascular disease, body mass index, mean systolic blood pressure of visits between 4 and 20 months, diabetes duration, hemoglobin A1c, total cholesterol, use of anti‐hypertensive medications, statin, and intensive glucose‐lowering therapy.

Abbreviations: CI, confidence interval; DSST, Digit Symbol Substitution Test; HR, hazard ratio; MMSE, Mini‐Mental State Examination; RAVLT, Rey Auditory Verbal Learning Test.

^a^ A score at the 20‐month or 40‐month follow‐up greater than 1 standard deviation (SD) below the baseline mean indicates cognitive decline.

^b^ A score at the 20‐month or 40‐month follow‐up more than 1 SD above the baseline mean indicates cognitive decline.

### 3.3 Associations of systolic BPV with change in MRI parameters

At 40 months, participants showed a significant decrease in TBV, gray matter volume, and a significant increase in white matter lesion volume (Table 2). There was a significant and positive correlation between systolic BPV and change in white matter lesion volume over 40 months after adjustment for age, sex, and days since randomization (*β* = 0.09, 95% CI 0.03–0.15; Table 4). It remained significant after further adjustment for an additional number of confounding factors (*β* = 0.07, 95% CI 0.01–0.13). No significant associations were observed between systolic BPV and changes in TBV, gray matter volume, or white matter volume over 40 months.

**TABLE 4:** Associations of systolic blood pressure variability levels with changes in brain magnetic resonance imaging parameters.

	Model 1		Model 2	
	*β* (95% CI)	*p* Value	*β* (95% CI)	*p* Value
Change in total brain volume	−0.68 (−2.38, 1.02)	0.436	−0.30 (−1.95, 1.35)	0.727
Change in gray matter volume	−0.51 (−1.40, 0.38)	0.263	−0.26 (−1.13, 0.62)	0.572
Change in white matter volume	−0.02 (−1.03, 0.99)	0.968	0.09 (−0.90, 1.07)	0.865
Change in white matter lesion volume	0.09 (0.03, 0.15)	0.003	0.07 (0.01, 0.13)	0.020

*Note*: Model 1 was adjusted for age, sex, and days since randomization, with random effects for participant. Model 2 was adjusted for age, sex, race, education, current smoking status, alcohol drinking status, history of cardiovascular disease, body mass index, mean systolic blood pressure of visits between 4 and 20 months, diabetes duration, hemoglobin A1c, total cholesterol, use of anti‐hypertensive medications, statin, intensive glucose‐lowering therapy, and days since randomization, with random effects for participant.

Abbreviation: CI, confidence interval.

## 4 DISCUSSION

The current study provided new evidence that a higher visit‐to‐visit systolic BPV was associated with future cognitive decline in persons with type 2 diabetes. We found that higher levels of systolic BPV, independent of mean systolic BP, were associated with a greater decline in psychomotor function and working memory speed evaluated by the DSST. Additionally, higher levels of systolic BPV were associated with the progression of white matter lesion over 40 months evaluated by the brain MRI in patients with type 2 diabetes.

Previous studies have revealed systolic BPV as a risk factor for cognitive decline in the general population.^26^, ^27^, ^28^ However, less is known in patients with diabetes. In addition, previous studies did not report findings for a specific cognitive domain. The mechanism connecting systolic BPV with cognitive decline is not well understood. Cognitive decline can be affected by vascular and degenerative mechanisms.^29^ Increased BPV can cause hemodynamic instability, resulting in an elevated shear stress. This elevated stress may directly contribute to the development of small vessel disease and cerebral hypoperfusion,^4^ ultimately leading to neuronal cell injury.^2^ Furthermore, an elevated BPV might also indicate arterial stiffness, a condition associated with cognitive decline. The association arises from arterial stiffness amplifying sporadic fluctuations in BP.^30^ Accordingly, neuronal damage and cellular death may affect sections of the brain that are vulnerable, such as the subcortical white matter and the hippocampal region.^31^ Studies have shown that BPV may be responsible for cerebral small vessel diseases, including white matter lesions, microbleeds, silent infarctions, and brain atrophy, which contribute to cognitive impairment and dementia.^4^, ^6^, ^31^ A study in an elderly population cohort without dementia reported that increases in BP fluctuations across three visits over a 24‐month period were correlated with a greater extent of white matter hyperintensities and a higher incidence of silent infarcts.^32^ BPV may be a causative factor of the alterations in brain structure and function, which in turn might lead to cognitive decline. Further studies are needed to elucidate the pathogenesis of increased BPV in the development of cognitive decline in patients with type 2 diabetes.

Recent studies have shown that calcium channel blockers and non‐loop diuretics can reduce variability in BP from visit to visit in addition to BP level reduction.^33^ Furthermore, it has been demonstrated in experimental animals that antihypertensive treatment leads to a reduction in BPV, which independently contributes to the protection of organs.^34^ However, it is currently lacking empirical evidence to support the notion that BPV reduction enhances cardiovascular risk mitigation or cognitive function in human participants. Additional research is warranted to investigate the efficacy of interventions aimed at mitigating BPV in individuals diagnosed with type 2 diabetes, with regard to their potential to diminish the likelihood of cognitive decline and the onset of dementia.

This study utilized high‐quality data from the ACCORD‐MIND substudy with a large sample size. However, it has several limitations. First, cognitive decline is a slow process, and 40 months of follow‐up may be an inadequate duration of time to ascertain subtle differences in cognitive function.^35^ Second, individuals with HbA1c below 7.5%, and individuals with HbA1c above 7.5% but without history of or risk factors for CVD were excluded from ACCORD. Third, because patients volunteered to participate in MRI, there may be a selection bias in the analysis of associations between systolic BPV and brain MRI imaging parameters.

## 5 CONCLUSIONS

This post hoc analysis of data from the ACCORD‐MIND substudy highlights the increased risk of cognitive decline and the increased white matter lesion volume in association with increased systolic BPV in individuals with type 2 diabetes. To reduce systolic BPV during BP management in diabetes patients might be important in the prevention of cognitive decline. Additional studies are needed to explore effective strategies to reduce systolic BPV and to determine whether this can effectively lower the risk of cognitive decline in individuals with type 2 diabetes.

## AUTHOR CONTRIBUTIONS

*Conceptualization, formal analysis, methodology and writing—original draft*: Junmin Chen, Xuan Zhao, and Huidan Liu. *Investigation and writing—review and editing*: Kan Wang, Xiaoli Xu, Siyu Wang, Mian Li, and Ruizhi Zheng. *Conceptualization, supervision and writing—review and editing*: Libin Zhou, Yufang Bi, and Yu Xu. All authors contributed to the article and approved the submitted version.

## CONFLICT OF INTEREST STATEMENT

The authors declare no conflicts of interest. Yufang Bi is an Editorial Board member of Journal of Diabetes and a co‐author of this article. To minimize bias, she was excluded from all editorial decision‐making related to the acceptance of this article for publication.

## References

[1] Prince M , Guerchet M , Prina M. The Global Impact of Dementia 2013–2050. Alzheimer's Disease International; 2013.

[2] Kalaria RN . Cerebrovascular disease and mechanisms of cognitive impairment: evidence from clinicopathological studies in humans. Stroke. 2012;43(9):2526‐2534. 22879100 10.1161/STROKEAHA.112.655803

[3] Lattanzi S , Luzzi S , Provinciali L , Silvestrini M . Blood pressure variability in Alzheimer's disease and frontotemporal dementia: the effect on the rate of cognitive decline. J Alzheimers Dis. 2015;45(2):387‐394. 25790932 10.3233/JAD-142532

[4] Havlik RJ , Foley DJ , Sayer B , Masaki K , White L , Launer LJ . Variability in midlife systolic blood pressure is related to late‐life brain white matter lesions: the Honolulu‐Asia Aging study. Stroke. 2002;33(1):26‐30. 11779884 10.1161/hs0102.101890

[5] Rothwell PM , Howard SC , Dolan E , et al. Prognostic significance of visit‐to‐visit variability, maximum systolic blood pressure, and episodic hypertension. Lancet. 2010;375(9718):895‐905. 20226988 10.1016/S0140-6736(10)60308-X

[6] Sabayan B , Wijsman LW , Foster‐Dingley JC , et al. Association of visit‐to‐visit variability in blood pressure with cognitive function in old age: prospective cohort study. BMJ. 2013;347:f4600. 23900315 10.1136/bmj.f4600

[7] Frisoni GB , Fox NC , Jack CR , Scheltens P , Thompson PM . The clinical use of structural MRI in Alzheimer disease. Nat Rev Neurol. 2010;6(2):67‐77. 20139996 10.1038/nrneurol.2009.215 PMC2938772

[8] Saczynski JS , Siggurdsson S , Jonsson PV , et al. Glycemic status and brain injury in older individuals: the age gene/environment susceptibility‐Reykjavik study. Diabetes Care. 2009;32(9):1608‐1613. 19509008 10.2337/dc08-2300 PMC2732166

[9] van Harten B , de Leeuw F‐E , Weinstein HC , Scheltens P , Biessels GJ . Brain imaging in patients with diabetes: a systematic review. Diabetes Care. 2006;29(11):2539‐2548. 17065699 10.2337/dc06-1637

[10] Jack CR , Wiste HJ , Vemuri P , et al. Brain beta‐amyloid measures and magnetic resonance imaging atrophy both predict time‐to‐progression from mild cognitive impairment to Alzheimer's disease. Brain. 2010;133(11):3336‐3348. 20935035 10.1093/brain/awq277 PMC2965425

[11] Debette S , Markus HS . The clinical importance of white matter hyperintensities on brain magnetic resonance imaging: systematic review and meta‐analysis. BMJ. 2010;341:c3666. 20660506 10.1136/bmj.c3666 PMC2910261

[12] Manschot SM , Biessels GJ , de Valk H , et al. Metabolic and vascular determinants of impaired cognitive performance and abnormalities on brain magnetic resonance imaging in patients with type 2 diabetes. Diabetologia. 2007;50(11):2388‐2397. 17764005 10.1007/s00125-007-0792-z PMC2039826

[13] Bencivenga L , De Souto Barreto P , Rolland Y , et al. Blood pressure variability: a potential marker of aging. Ageing Res Rev. 2022;80:101677. 35738476 10.1016/j.arr.2022.101677

[14] Luo A , Xie Z , Wang Y , et al. Type 2 diabetes mellitus‐associated cognitive dysfunction: advances in potential mechanisms and therapies. Neurosci Biobehav Rev. 2022;137:104642. 35367221 10.1016/j.neubiorev.2022.104642

[15] Goff DC , Gerstein HC , Ginsberg HN , et al. Prevention of cardiovascular disease in persons with type 2 diabetes mellitus: current knowledge and rationale for the action to control cardiovascular risk in diabetes (ACCORD) trial. Am J Cardiol. 2007;99(12A):S4‐S20. 10.1016/j.amjcard.2007.03.002 17599424

[16] Williamson JD , Miller ME , Bryan RN , et al. The action to control cardiovascular risk in diabetes memory in diabetes study (ACCORD‐MIND): rationale, design, and methods. Am J Cardiol. 2007;99(12A):112i‐122i. 10.1016/j.amjcard.2007.03.029 17599421

[17] Launer LJ , Miller ME , Williamson JD , et al. Effects of intensive glucose lowering on brain structure and function in people with type 2 diabetes (ACCORD MIND): a randomised open‐label substudy. Lancet Neurol. 2011;10(11):969‐977. 21958949 10.1016/S1474-4422(11)70188-0 PMC3333485

[18] Folstein MF , Folstein SE , McHugh PR . “Mini‐mental state”: a practical method for grading the cognitive state of patients for the clinician. J Psychiatr Res. 1975;12(3):189‐198. 1202204 10.1016/0022-3956(75)90026-6

[19] Wechsler D, ed. *Wechsler Adult Intelligence Scale‐Revised*. Psychological Corporation; 1988.

[20] Lezak MD . Neuropsychological Assessment. Oxford University Press; 2004.

[21] Houx PJ , Jolles J , Vreeling FW . Stroop interference: aging effects assessed with the Stroop Color‐Word Test. Exp Aging Res. 1993;19(3):209‐224. 8223823 10.1080/03610739308253934

[22] Goldszal AF , Davatzikos C , Pham DL , Yan MXH , Bryan RN , Resnick SM . An image‐processing system for qualitative and quantitative volumetric analysis of brain images. J Comput Assist Tomogr. 1998;22(5):827‐837. 9754125 10.1097/00004728-199809000-00030

[23] Lao Z , Shen D , Liu D , et al. Computer‐assisted segmentation of white matter lesions in 3D MR images using support vector machine. Acad Radiol. 2008;15(3):300‐313. 18280928 10.1016/j.acra.2007.10.012 PMC2528894

[24] Price R , Allison J , Clarke G , et al. Magnetic resonance imaging quality control manual. 2015. Accessed September 13, 2024. https://www.acr.org/‐/media/ACR/Files/Clinical‐Resources/QC‐Manuals/MR_QCManual.pdf

[25] Anand SS , Friedrich MG , Lee DS , et al. Evaluation of adiposity and cognitive function in adults. JAMA Netw Open. 2022;5(2):e2146324. 35103790 10.1001/jamanetworkopen.2021.46324 PMC8808326

[26] Jia P , Lee HWY , Chan JYC , Yiu KKL , Tsoi KKF . Long‐term blood pressure variability increases risks of dementia and cognitive decline: a meta‐analysis of longitudinal studies. Hypertension. 2021;78(4):996‐1004. 34397274 10.1161/HYPERTENSIONAHA.121.17788

[27] Mahinrad S , Bennett DA , Sorond FA , Gorelick PB . Blood pressure variability, dementia, and role of antihypertensive medications in older adults. Alzheimers Dement. 2023;19(7):2966‐2974. 36656086 10.1002/alz.12935 PMC10354219

[28] Ma Y , Blacker D , Viswanathan A , et al. Visit‐to‐visit blood pressure variability, neuropathology, and cognitive decline. Neurology. 2021;96(23):e2812‐e2823. 33903194 10.1212/WNL.0000000000012065 PMC8205457

[29] Iadecola C , Duering M , Hachinski V , et al. Vascular cognitive impairment and dementia: JACC scientific expert panel. J Am Coll Cardiol. 2019;73(25):3326‐3344. 31248555 10.1016/j.jacc.2019.04.034 PMC6719789

[30] Rabkin SW . Arterial stiffness: detection and consequences in cognitive impairment and dementia of the elderly. J Alzheimers Dis. 2012;32(3):541‐549. 22886012 10.3233/JAD-2012-120757

[31] Iadecola C . Neurovascular regulation in the normal brain and in Alzheimer's disease. Nat Rev Neurosci. 2004;5(5):347‐360. 15100718 10.1038/nrn1387

[32] Brickman AM , Reitz C , Luchsinger JA , et al. Long‐term blood pressure fluctuation and cerebrovascular disease in an elderly cohort. Arch Neurol. 2010;67(5):564‐569. 20457955 10.1001/archneurol.2010.70 PMC2917204

[33] Webb AJS , Fischer U , Mehta Z , Rothwell PM . Effects of antihypertensive‐drug class on interindividual variation in blood pressure and risk of stroke: a systematic review and meta‐analysis. Lancet. 2010;375(9718):906‐915. 20226989 10.1016/S0140-6736(10)60235-8

[34] Xie H‐H , Zhang X‐F , Chen Y‐Y , Shen F‐M , Su D‐F . Synergism of hydrochlorothiazide and nifedipine on blood pressure variability reduction and organ protection in spontaneously hypertensive rats. Hypertens Res. 2008;31(4):685‐691. 18633181 10.1291/hypres.31.685

[35] Bateman RJ , Xiong C , Benzinger TLS , et al. Clinical and biomarker changes in dominantly inherited Alzheimer's disease. N Engl J Med. 2012;367(9):795‐804. 22784036 10.1056/NEJMoa1202753 PMC3474597

